# Transfer of skills acquired through Cognitive Orientation to daily Occupational Performance (CO‐OP) approach in children with executive functions deficits following acquired brain injury

**DOI:** 10.1111/1440-1630.70040

**Published:** 2025-07-30

**Authors:** Clarisse Vezinat, Hélène Lebrault, Hugo Câmara‐Costa, Rose Martini, Mathilde Chevignard

**Affiliations:** ^1^ Rehabilitation Department for Children with Acquired Neurological Injury Paris‐Est Val‐de‐Marne Hospitals Saint Maurice France; ^2^ Faculty of Medicine Sorbonne University Paris France; ^3^ CNRS, INSERM, Laboratoire d'Imagerie Biomédicale, LIB Sorbonne Université Paris France; ^4^ GRC 24, Handicap Moteur et Cognitif et Réadaptation (HaMCRe) Sorbonne Université Paris France; ^5^ Occupational Therapy Program, School of Rehabilitation Sciences, Faculty of Health Sciences University of Ottawa Ottawa Ontario Canada

**Keywords:** acquired brain injury, child, Cognitive Orientation to daily Occupational Performance (CO‐OP) approach, executive functions, occupational therapy, transfer

## Abstract

**Introduction:**

Children with acquired brain injury (ABI) often have executive function (EF) deficits that affect their ability to carry out activities efficiently. Cognitive Orientation to daily Occupational Performance (CO‐OP) is a cognitive approach to problem‐solving. It has demonstrated effectiveness in a variety of populations, most recently with children with EF deficits following ABI. One of the challenges of rehabilitation is transferring skills acquired during sessions into everyday life. The literature suggests that CO‐OP enables transfer of skills to untrained tasks, but research on transfer following its use with children with ABI is limited. The aims of this study were (1) to determine whether skills acquired during CO‐OP transfer to new untrained activities, (2) to study the possible influence of parental involvement in CO‐OP on children's attainment of transfer goals, and (3) to determine if the degree of difference between trained and untrained goals is associated with achievement of transfer.

**Methods:**

A quasi‐experimental design was used. Eleven children with EF deficits following ABI took part in the study. At the end of CO‐OP intervention, children identified three new goals. These ‘transfer’ goals were assessed at baseline (immediately post‐intervention) and at 2‐, 4‐ and 6‐month, using the Goal Attainment Scale (GAS) and the self‐ and parent‐rated Canadian Occupational Performance Measure (COPM). Children did not receive any specific intervention during this time.

**Consumer and Community Involvement:**

There was no consumer and community involvement.

**Results:**

At 6 months, GAS indicated that 22 of 32 transfer goals had improved by at least one level. COPM showed a significant increase in (1) child perception of performance and satisfaction for 24/31 and 25/31 goals, respectively, and (2) parent perception of performance and satisfaction for 19/29 of goals.

**Conclusion:**

This study showed that CO‐OP facilitates transfer of acquired skills to new, untrained activities, even after the end of the intervention.

**Key Points for Occupational Therapy**

• To enable meaningful participation, it is important to ensure that learning acquired in rehabilitation is transferred to everyday life.
• CO‐OP facilitates the transfer of acquired skills to new, untrained activities, after the end of the intervention.
• Parent involvement appears to influence the progression of untrained transfer goals.

## 1 INTRODUCTION

Acquired brain injury (ABI) in childhood and adolescence can occur as a result of traumatic (e.g. fall, motor vehicle accident, and assault) or non‐traumatic brain lesions (e.g. most commonly stroke, tumour or infection). ABI is the leading cause of death and acquired disability in children (V. Anderson et al., 2005; McKinlay et al., 2008). Children who sustained moderate to severe ABI (i.e. at risk for long‐lasting problems as per Tenovuo et al., 2021) may not only experience significant motor and sensory impairments but also adverse effects on behaviour and cognition, particularly executive functions (EFs) (V. Anderson & Catroppa, 2006; Krasny‐Pacini et al., 2017). EFs are defined as a set of independent but interrelated high‐level cognitive processes for producing adaptive, goal‐directed intentional behaviour during a non‐routine situation (Diamond, 2013). They encompass several processes, including inhibition and working memory, flexibility, planning, as well as anticipation, activity initiation, self‐regulation, deployment of attention, and feedback use (P. Anderson, 2002). EFs develop throughout childhood and adolescence and play an important part in new learning, behaviour regulation, emotional control, social interaction, and academic achievement (P. Anderson, 2002). These difficulties are likely to have a more pronounced effect on children, a time of accelerated EF growth (Keenan et al., 2021). Impaired EFs compromise the child's ability to learn and acquire new skills, making it more challenging for them to acquire age‐appropriate skills across all areas and tasks of everyday living (Galvin & Mandalis, 2009). Therefore, deficits in EF generally have a significant impact on the occupational performance of children with ABI, that is, performance in carrying out daily activities, including their participation in learning and other school activities (Chevignard et al., 2010).

Traditional cognitive rehabilitation methods used with children have mainly focussed on remediating the impairments underlying the difficulties of daily life, rather than on the activity performance itself (Polatajko & Mandich, 2004). These methods have shown little effectiveness in improving performance in the person's daily activities (Krasny‐Pacini, Limond, & Chevignard, 2016). The results of a systematic review of paediatric occupational therapy approaches support the use of activity‐level over impairment‐focussed approaches (Novak & Honan, 2019). It is therefore essential to focus rehabilitation on more ecological, that is, task‐oriented approaches, so that children can acquire new skills to perform their activities, and subsequently, ideally, transfer these newly acquired skills to new activities within their daily environment (Camm et al., 2021; Geusgens et al., 2007; Laatsch et al., 2020).

In the literature, the concept of transfer is defined in different ways. Generally, authors agree that transfer is the application of skills and/or strategies to learning a new task: for example, applying the strategies and skills learned while skiing to learn how to snowboard (Geusgens et al., 2007; Müssgens & Ullén, 2015). While the performance of a skill in a different context is also viewed as transfer in the motor learning literature, Polatajko and Mandich (2004) differentiate this transfer different context as generalisation, that is, the application of a specific skill learned in a different context, for example, riding a bicycle in one's backyard to riding it on the street. However, the concepts of transfer and generalisation are often difficult to dissociate, as they frequently occur simultaneously (Geusgens et al., 2007; McEwen et al., 2015; Polatajko & Mandich, 2004).

The systematic review by Laatsch et al. (2020), which sought to identify effective cognitive and behavioural rehabilitation interventions for children with ABI, confirm that cognitive rehabilitation approaches targeting impairments underlying the daily life difficulties are not highly effective in improving skills in those everyday tasks. In this review, the effective interventions largely targeted family and caregivers to take on the role of ‘reminder’ in the person's everyday environment, thereby ensuring a generalisation of the strategies that were learned. Similarly, Camm et al.'s (2021) systematic review on cognitive interventions for children with ABI highlights the importance of involving family when aiming to facilitate transfer and generalisation of learned skills to the home and school environment.

Cognitive Orientation to daily Occupational Performance (CO‐OP) is a metacognitive problem‐solving approach that stems from cognitive, cognitive–behavioural, and motor learning theories (Polatajko & Mandich, 2004). It is a client‐centred intervention, whereby the person is considered an actor in his/her intervention. As such, the goals trained during the intervention are chosen by the children. In CO‐OP, children are asked to identify three occupational performance goals related to their difficulties in their daily life activities. Then, using a guided discovery process, the therapist guides children through an iterative problem‐solving process, framed by metacognitive strategy of Goal‐Plan‐Do‐Check enabling them to attain the activity goals they had set (Polatajko & Mandich, 2004).

The CO‐OP approach has four objectives: (1) skill acquisition, (2) strategies use, (3) generalisation of learning, and (4) transfer of learning (Polatajko & Mandich, 2004). This intervention approach specifically addresses the issue of generalisation and transfer (McEwen et al., 2015; Polatajko & Mandich, 2004). Indeed, the therapist is expected to create opportunities for children to practice or apply what they learned during the intervention session to other contexts and/or tasks. These intentional opportunities not only increase their practice of learned skills in a different context but also explicitly draw their attention to the use of previously learned strategies to the performance of new activities. Parents are also invited to become involved in the CO‐OP process, for instance, cueing the child to implement learned strategies at home (Polatajko & Mandich, 2004).

Several studies suggest that CO‐OP effectively facilitates the transfer of acquired skills to new tasks in various populations, including children with developmental coordination disorder (Capistran & Martini, 2016; Houldin et al., 2018), adults following stroke or traumatic brain injury (Dawson et al., 2009; McEwen et al., 2015), and, more recently, children with cerebral palsy or spina bifida (Öhrvall et al., 2023). To our knowledge, few CO‐OP studies have involved children with cognitive deficits following ABI (Hunt et al., 2019; Jackman et al., 2018; Lebrault et al., 2021, 2023; Missiuna et al., 2010). Missiuna et al., Hunt et al. and Jackman et al. studied the effectiveness of CO‐OP on performance in daily activities in children with ABI. Although Hunt et al. (2019) suggested that children transferred their learning (using untrained control goals), overall, those three studies did not (1) specifically take into account EF deficits, (2) focus specifically on the measurement of presence of transfer (Hunt et al., 2019) or did not study transfer (Jackman et al., 2018; Missiuna et al., 2010). No study to date has specifically explored whether the CO‐OP approach may also facilitate the transfer of acquired skills in children with EF following an ABI. Such a study is needed, given the role EF has in skill acquisition and transfer.

Lebrault et al. (2021) conducted a pilot study using a single‐case experimental design methodology, exploring the use of a CO‐OP intervention with children with EF deficits following an ABI. Results showed improvement in the occupational performance of selected daily life tasks in these children and also suggested a certain degree of transfer of the skills to the children's untrained, control goal. A second study, using a replicated single‐case experimental design with randomised multiple baselines, across children and across goals, was carried out to confirm the pilot study results with a larger sample of children. Results of the Goal Attainment Scale (GAS) showed that overall, children improved in 26 of the 35 trained goals (Lebrault et al., 2023).

Given the limited research on the transfer of learning following the use of the CO‐OP approach with children with ABI, the aims of the current study were (1) to determine whether children demonstrated the transfer of skills to new tasks (goals) following a CO‐OP intervention, (2) to study the possible influence of the involvement of parents in CO‐OP on children's attainment of transfer goals, and (3) to determine if the degree of difference in task characteristics, between trained and untrained transfer goals, is associated with the achievement of transfer.

## 2 METHODS

This study was part of a larger study that used a single‐case experimental design (SCED) methodology to assess the effectiveness of CO‐OP to improve occupational performance in children/youth with EF deficits following severe ABI (Lebrault et al., 2023) Ethic approval was obtained from the Sud‐Ouest et Outre‐Mer II ethics committee and registered on ClinicalTrials.gov (NCT04560777).

### 2.1 Participants

The current study took place in two large city rehabilitation centres for children, youth, and young adults.

Children were included in the study if they met the following criteria: (1) youth aged between 8 and 21 years with a diagnosis of ABI, sustained at least 6 months previously; (2) still attending an inpatient or outpatient rehabilitation programme, including multidisciplinary rehabilitation and specialised on‐site schooling; (3) evidence of a dysexecutive syndrome on the neuropsychological assessment, including three subtests of the Behavioural Assessment of the Dysexecutive Syndrome (BADS‐C for 8–15 years, BADS if ≥16 years) (Emslie et al., 2003; Wilson et al., 1996), the Children's Cooking Task (Chevignard et al., 2010), and the Behaviour Rating Inventory of Executive Function (BRIEF) questionnaire (Roy et al., 2011); (4) vision and hearing normal or sufficient to communicate effectively (with appropriate correction if necessary); and (5) sufficient speaking and comprehension skills to communicate effectively and accurately. Exclusion criteria were (1) non‐French‐speaking children and/or parents; (2) cognitive/language deficit incompatible with understanding and participating in therapy (as per clinical or parent report); (3) children not able to identify at least four occupational performance goals (i.e. activities and tasks they wanted or needed to do but were not able to do); (4) neurological, psychiatric, genetic or learning disorder diagnosed prior to the occurrence of the ABI (in order not to confound results); (5) diagnosed severe anxiety and/or depressive disorder, incompatible with participation in the study; and (6) inability to commit until the end of the study.

Twelve youths aged 8 to 16 years with EF deficits following severe ABI met these criteria and participated in the larger single subject design study on the effectiveness of the CO‐OP approach (Lebrault et al., 2023).

**FIGURE 1:**
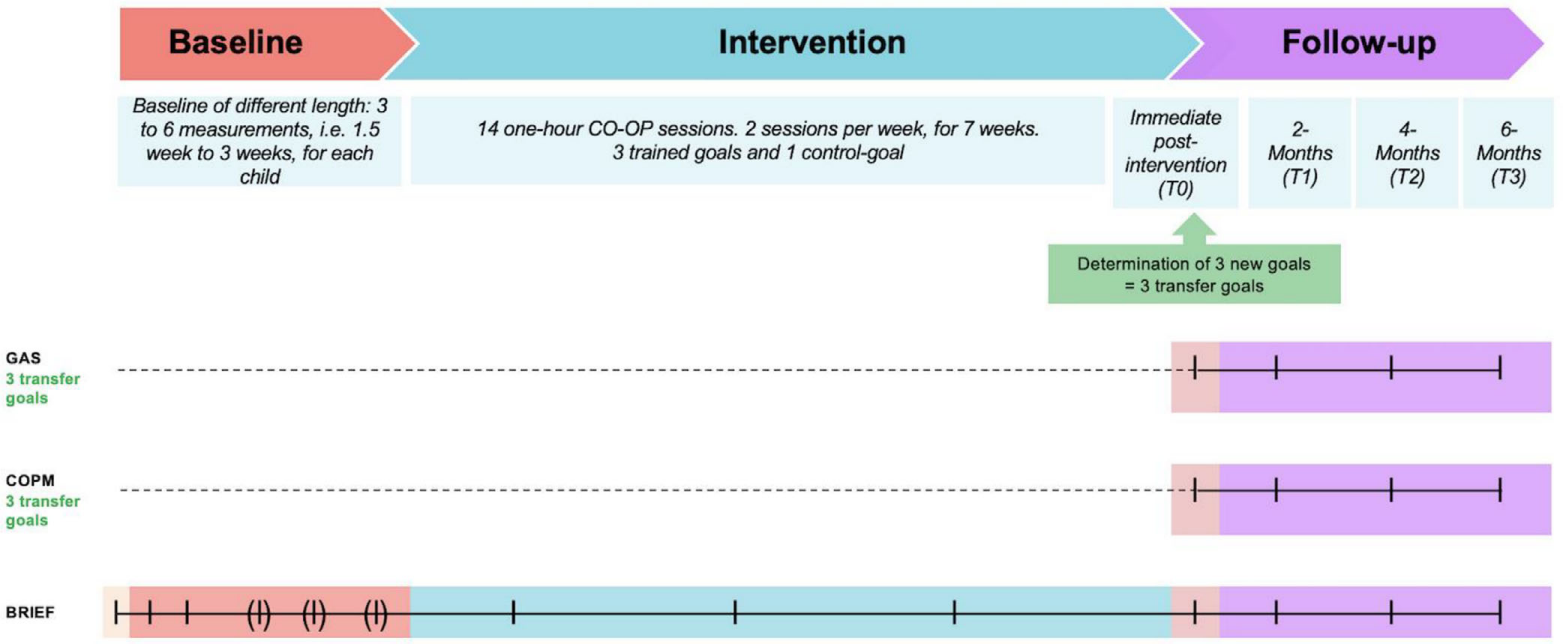
Procedure chart and measures of transfer.

Figure 1 provides an overview of the study procedure and times at which each of the measures was applied.

### 2.2 Positionality statement

This study's research team comprised individuals with multidisciplinary professional backgrounds: occupational therapy, rehabilitation medicine, and psychology. All authors holding advanced degrees at the Master's or PhD level, and they are all researchers and therapists (except for one) in the field of paediatric rehabilitation. The authors have a commitment to improving the performance in daily activities of children with EF following ABI and pose collective diversity of experiences as both clinicians and researchers in their respective fields.

### 2.3 Measures

The following measures were used to collect the data for this study:

#### 2.3.1 The Canadian Occupational Performance Measure (COPM)

The COPM (Law et al., 2019) is a semi‐structured interview designed to help individuals identify occupational performance issues they would like to work on in therapy (so as to establish therapy goals), and to measure changes in perceived performance and satisfaction in those targeted tasks. Importance, perceived performance, and satisfaction are rated on a respective 10‐point scale. The COPM has adequate to high test–retest, good content, and criterion validity (Law et al., 2019). This standardised tool was also of interest to the study because it is often used in clinical practice and research in paediatric rehabilitation (Cusick et al., 2006). A 2‐point change between two assessments is considered an important change (McColl et al., 2023). The COPM was also used to identify the three new goals that would serve as the ‘untrained transfer goals’ at the end of the CO‐OP intervention. Then, for each new transfer goal identified immediately post‐intervention, four different COPM ratings were obtained: (1) the children's perceived occupational performance rating, (2) the children's satisfaction rating, (3) the parent's perceived occupational performance rating, and (4) the parent's satisfaction rating. This was performed again at each follow‐up time point (2‐, 4‐, and 6‐months post‐intervention).

#### 2.3.2 The Goal Attainment Scale (GAS)

The GAS was used to monitor children's progress in transfer goals performance over time. The use of the GAS to determine effectiveness of intervention is becoming more prevalent in both research and clinical physical and rehabilitation medicine practice (Bard‐Pondarré et al., 2023). This tool has value in research where standardised assessments are not available to measure the construct being explored (Krasny‐Pacini, Evans, et al., 2016). The use of the GAS also allows for performance assessment of personalised goals in more ecological settings. In this study, the five levels of GAS used are those defined according to Steenbeek's methodology (Steenbeek et al., 2010): −2 = initial level, −1 = presence of progress but goal not achieved, 0 = goal is achieved as expected (expected level at the end of the follow‐up phase), +1 = goal is achieved exceeding expectations, and +2 = the most favourable outcome.

In keeping with the philosophy of client‐centred practice, the GAS level criteria for each goal were defined in collaboration with the child following the identification of their three new transfer goals. To be used in a research setting, GAS requires a high methodological quality approach. For this reason, we used the GAS Quality Rating System, established by Pradeau et al. (2024), to ensure that the GAS had been written rigorously, thus establishing outcome measures that were valid and reliable. A blinded independent expert trained in the GAS methodology (i.e., who did not know the children, was not involved in defining GAS levels, and did not participate in the assessment nor in the intervention) validated the GAS scales produced by the rehabilitation team. This expert ensured that the methodological criteria for writing the GAS were met and suggested modifications when necessary. Appendix A provides an example of a GAS used.

To determine progress in the three transfer goals (which were not addressed in rehabilitation), children were videotaped performing each of their three transfer goals at four time points: upon goal identification (i.e. just post‐CO‐OP intervention) and again at 2‐, 4‐ and 6‐month post‐intervention). To limit the risk of bias, the video recordings were analysed in random order by evaluators trained in GAS methodology. These persons were independent from the study, did not know the children, and were blinded to the study phase. Furthermore, 20% of the goal performances were scored by a second rater using the GAS to ensure inter‐rater reliability (Kratochwill et al., 2013). This was carried out using weighted Cohen's Kappa coefficient (Landis & Koch, 1977), on the Dotmatics GraphPad (https://www.graphpad.com/quickcalcs/kappa2/ ). Reliability coefficients were interpreted as follows: fair (0.00 < *k <* 0.40), moderate (0.41 < *k* < 0.60), or strong agreement (*k* > 0.61) (Landis & Koch, 1977). The inter‐rater reliability of the GAS scores ranged from moderate to excellent/almost perfect.

#### 2.3.3 Generalisation and Transfer Scale (G&T scale)

The Generalisation and Transfer (G&T) scale was developed by Houldin (2018) to evaluate the degree of difference, or the level of generalisation and transfer, required to move between trained and untrained skills. While generalisation refers to applying what is learnt from one context to another, transfer refers to applying what is learnt from one pattern of skill performance to another (Houldin, 2018). The G&T scale assesses these two dimensions (context and pattern) by quantifying both contextual differences/similarities (associated with generalisation) and pattern differences/similarities (associated with transfer) between a pair of skills (reference and target skill). The trained skill (or goal) is considered as the reference skill, whereas the untrained skill (or goal) is referred to as the target skill.

Consistent with the view of the common elements theory (Thorndike, 2013) and of cognitive processes (Geusgens et al., 2007), it is anticipated that the higher the G&T score, the more different the activities and the less likely (or more difficult) for generalisation and transfer of skills between the two compared activities. Houldin (2018) reports this scale to have good content validity and inter‐rater reliability.

We used the G&T scale to quantify the degree of generalisation‐transfer inherent in the pair of trained goals during CO‐OP and the untrained new transfer goals. To do this, we scored the G&T scale (see Table 1 for example) between the three target skills (i.e. the three post‐intervention new transfer goals) and each of the three reference skills (i.e. in‐CO‐OP session trained goals). Inter‐rater reliability of the G&T was assessed for 20% of the G&T scores (for 24 pairs of goals) by two independent occupational therapists. Nearly perfect agreement was obtained: weighted Cohen's Kappa = 0.801 (95% CI: 0.626–0.976) (Parker & Vannest, 2009).

**TABLE 1:**
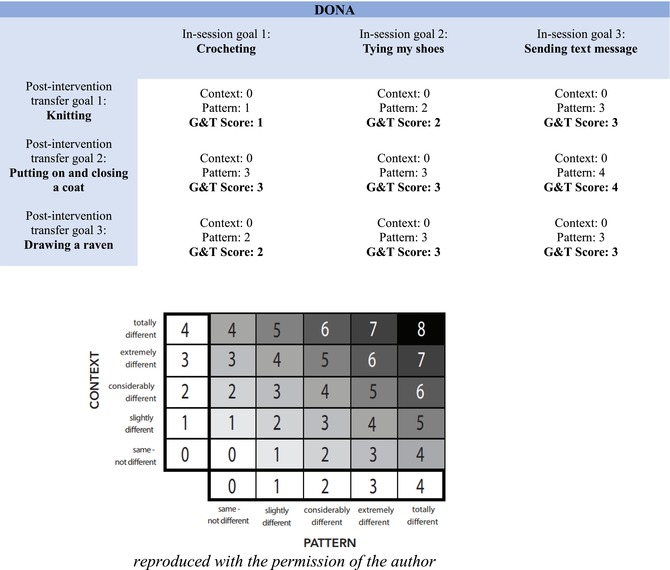
Example of a children's G&T scale.

#### 2.3.4 Measures of the involvement of parents in the CO‐OP process

In CO‐OP, the involvement of people in the child's environment (parents, other family members, teachers, care‐partners …) is encouraged to facilitate the process of skill learning and generalisation and transfer (Polatajko & Mandich, 2004). Involvement can occur in different ways at different degrees: for instance, assisting the child to complete the practice homework given by the therapist, reminding the child about the global strategy, or prompting the child to use strategies and skills learned at therapy sessions (Cameron et al., 2017). Parents' involvement has been shown to benefit the generalisability of skills leaned in therapy (Burrell & Borrego, 2012; Tetreault et al., 2003).

In the current study, we explored the association between the degree of parents' involvement with the evolution of children' GAS and COPM scores during follow‐up. The degree of parents' involvement was determined using (1) the therapists' notes on exchanges with parents between CO‐OP sessions (e.g. Were parents contactable? Were the occupational therapists able to report to them how the previous session had gone? Were the parents aware of the homework to be performed between sessions?…); (2) responses to a questionnaire on the use of CO‐OP proposed to parents during CO‐OP intervention at 2, 4‐ and 6‐intervention weeks (e.g. Have you practiced CO‐OP in the last 2 weeks? On what goals? How often?…); and (3) feedback from parents on their understanding of the approach and their own involvement during the CO‐OP intervention, gathered during an interview at the end of the CO‐OP intervention (Dietrich et al., 2024). The degree of parents' involvement was classified into two categories:

- *Medium to high involvement*: the notes of the occupational therapist who carried out the CO‐OP intervention and the answers to the questionnaire on the use of CO‐OP at home show regular exchanges with parents, and elements of these exchanges provided evidence that CO‐OP was practiced at home between sessions. The parents reporting around their CO‐OP experience showed a good understanding of the CO‐OP approach, an implementation of the approach in daily life, as well as an ecological context favouring its implementation, with support from the family members.
- *Poor or no involvement* : the notes of the occupational therapist who carried out the CO‐OP intervention and the answers to the questionnaire show little or no exchanges with the parents and poor or no evidence that the CO‐OP was put into practice at home between sessions. The analysis of the interviews shows a limited or poor understanding of the CO‐OP approach and poor or no implementation of the CO‐OP in daily life.

The inter‐rater reliability of categorisation of children according to the degree of involvement of parents was assessed for eight children by three independent occupational therapists. Agreement was perfect: weighted Cohen's Kappa = 1.0 (95% CI 1.0–1.0) (Landis & Koch, 1977).

### 2.4 Larger study protocol and procedure

The larger study protocol was a single subject design with a baseline and intervention phase (Lebrault et al., 2023). Prior to the beginning of the intervention (i.e. at the end of the baseline phase), the children identified four goals using the COPM. Three were trained, whereas one was left untrained and considered ‘control goal’. Achievement of the three trained goals and the control goal were rated twice a week throughout the baseline and intervention phases, then at immediate post‐intervention and at 2‐, 4‐, and 6‐month post‐intervention using the GAS. The CO‐OP intervention followed the key principles described in the original CO‐OP protocol (Polatajko & Mandich, 2004), with fourteen 45‐min sessions instead of the original 10, twice a week for 7 weeks. The increase in the number of sessions was determined based on the participants severe executive function profile and associated deficits, according to the findings of a previous study (Krasny‐Pacini et al., 2014) and the reviews by Camm et al. (2021) and Laatsch et al. (2020).

Each week, the therapist responsible for the child's CO‐OP sessions contacted the parents or the care partner to share information on how the sessions were going and on the homework to be carried out at home, that is, activities that fostered practice and the application of the strategies learned during the sessions. In addition, parents were invited to participate in their child's CO‐OP sessions (if feasible for them), to further encourage their involvement.

### 2.5 Study design for assessment of transfer

At the end of the CO‐OP intervention (i.e. end of the intervention phase), a new COPM interview was conducted with each of the children to identify three new issues (which became the transfer goals). These goals were not trained with CO‐OP nor during any other type of therapy session. The aim was to assess whether following the CO‐OP intervention, without any further intervention, improvement in the occupational performance of these non‐trained goals could be observed, and to explore factors that might be associated with this transfer. For this purpose, a quasi‐experimental repeated measures design without a control group was used. Quasi‐experimental research designs examine whether there is a causal relationship between independent and dependent variables (Rogers & Révész, 2020).

### 2.6 Data analysis

Statistical analyses were performed using SPSS software (v29.0.1.0; IBM Corp). We confirmed normality of the variables using Shapiro–Wilk tests (Shapiro & Wilk, 1965), and used descriptive statistics for parametric (Mean [*M*], standard deviation [SD]), non‐parametric (median and range [min–max]), and categorical variables (frequency [*n*] and percentages [%]).

We used graphical displays to perform visual analysis of the GAS and COPM scores of the transfer goals during follow‐up: immediate post‐intervention period (T0), and at 2‐month (T1), 4‐month (T2), and 6‐month (T3) post‐intervention. Subsequently, to determining whether occupational performance changed over time, we conducted non‐parametric Friedman tests (1937) to determine differences between T0 (when goals were identified) and T3 (6‐month follow‐up) in the GAS scores and difference in T0 − T3 in the COPM scores. The same procedure was used to analyse the evolution of GAS scores and COPM scores between T0 and T3 depending on the level of parental involvement.

We used Kendall rank (*τ*_b_) correlation procedures (1938) to investigate the potential association between progress in transfer goals (assessed with the GAS, observed between T0 and T3) and the degree of similarity between tasks as determined by the G&T scores. The aim was to assess whether the children who had made the most progress in achieving their transfer goals (target skills) were also those whose transfer goals had the lowest score on the G&T scale; in other words, we wanted to know whether transfer goals showed more progress or were more easily achieved when they were more similar (in terms of context of achievement and pattern) to those trained during CO‐OP. Effect sizes were calculated, and their magnitude was interpreted as absent (*τ*_b_ = 0), small (0 < *τ*_b_ ≤ 0.29), medium (0.3 ≤ *τ*_b_ ≤ 0.49, or large (*τ*_b_ > 0.5). Three correlations were performed, as summarised in Table 2.

**TABLE 2:** Analysis of the potential association between progress in transfer goals (assessed with the GAS; observed between immediate post‐intervention, T0; and 6‐month post‐intervention, T3) and the degree of similarity between tasks as determined by the G&T score.

	G&T scores *New transfer goal/trained goal 1*	AND	Progress in transfer goals: *T3* − *T0 new transfer goal GAS scores*	Then …
**Correlation 1**	Example of Dona: ○ Score 1: cutting/crocheting ○ Score 2: knitting/crocheting ○ Score 3: coat/crocheting	**AND**	Example of Dona: ○ (T3 − T0) for the transfer goal 1: knitting ○ (T3 − T0) for the transfer goal 2: Putting on and closing a coat ○ (T3 − T0) for the transfer goal 3: drawing	The statistical software proceeds in this way for the 11 children, compiles the scores, and calculates the correlation coefficient

	G&T scores *New transfer goal/trained goal 2*	AND	Progress in transfer goals: *T3* − *T0 new transfer goal GAS scores*	Then …
**Correlation 2**	Example of Dona: ○ Score 1: cutting/tying my shoelaces ○ Score 2: knitting/tying my shoelaces ○ Score 3: coat/tying my shoelaces	**AND**	Example of Dona: ○ (T3 − T0) for the transfer goal 1: knitting ○ (T3 − T0) for the transfer goal 2: Putting on and closing a coat ○ (T3 − T0) for the transfer goal 3: Drawing	The statistical software proceeds in this way for the 11 children, compiles the scores, and calculates the correlation coefficient

	G&T scores *New transfer goal/trained goal 3*	AND	Progress in transfer goals: *T3* − *T0 new transfer goal GAS scores*	Then …
**Correlation 3**	Example of Dona: ○ Score 1 cutting/sending text messages ○ Score 2 knitting/sending text messages ○ Score 3 coat/sending text messages	**AND**	Example of Dona: ○ (T3 − T0) for the transfer goal 1: knitting ○ (T3 − T0) for the transfer goal 2: putting on and closing a coat ○ (T3 − T0) for the transfer goal 3: drawing	The statistical software proceeds in this way for the 11 children, compiles the scores, and calculates the correlation coefficient

## 3 RESULTS

Eleven of the 12 children participated in the study until 6‐month follow‐up (the 12th children participated in the intervention study until post‐test but did not wish to continue after the CO‐OP intervention). Their characteristics are summarised in Table 3. Children's names have been changed to respect their anonymity. While all children completed the 14 CO‐OP intervention sessions and follow‐up sessions, scores are missing for two children: GAS and COPM scores for Nathan's third transfer goal (skate‐boarding could not be tested because of medical contraindications, following surgical recovery of a cranial flap); parents COPM' scores for Karl's transfer goals (parents were unreachable due to summer vacation).

**TABLE 3:** Participants characteristics.

ORIGINAL SCED				
	Nathan	Karl	Ben	Dona
**Gender and age at time of inclusion (years, months)**	Boy – 10y 10 m	Boy – 9y 10 m	Boy – 14y	Girl – 13y 1 m
**Family structure**	Parents divorced	Parents divorced	Parents divorced	Two‐parent household
**Siblings**	2	3	1	3
**Parent's education level** Number of years of study after graduation from high school	2 (mother); 0 (father	0 (father and mother)	2 (mother); 2 (stepfather)	5 (mother); 2 (father)
**Type of injury/diagnosis**	Severe traumatic brain injury (fall, 9 m)	Severe traumatic brain injury (gunshot wound)	Severe traumatic brain injury (motor vehicle accident)	Left hemispherotomy in the context of a Rasmussen syndrome
**Glasgow coma scale (+ duration of coma in days)** 13–15: mild; 9–12: moderate; 3–8: severe	8 (15)	6 (5)	3 (13)	N/A
**GOS‐E Peds**	Severe disability: upper level	Severe disability: lower level	Severe disability: lower level	Severe disability: lower level
**Associated impairments**	Word finding difficulties; fine motor impairment (distal tremors)	Right hemiparesis in a left‐handed boy; amblyopia of the right eye; alexia; agraphia; moderate aphasia	Cerebellar syndrome; delayed consolidation of the right tibia fracture; slow processing speed, overall slowness of execution	Right hemiparesis in a right‐handed girl; cerebellar syndrome; visual–spatial and constructive difficulties; aphasia
**Time since injury at time of inclusion (months)**	7 months	26 months	7 months	20 months
**Schooling within rehabilitation department**	Specialised classroom with a small number of students	Specialised classroom with a small number of students	Specialised classroom with a small number of students	Specialised classroom with a small number of students
**BADS‐C** Standard scores; [mean (SD) 10 (3)]				
Six parts test	7	7	4	4
Zoo part 1	5	10	7	7
Zoo part 2	7	6	4	4
**Children's cooking task**				
Number of errors/*z* score (compared to age‐matched controls)	54/−2.35	64/−3.3	134/−3.9	129/−9.3
Task duration (min)/*z* score (compared to age‐matched controls)	29/0.5	41/−0.6	68/−4.3	95/−7.5
**BRIEF questionnaire (before study start)** Global executive composite score (GEC) *T*‐score, (mean [SD] 50 [10]); clinical range cut‐off: *T*‐score ≥ 65				
Parents rating	53	70 (educator's rating)	71	58
Teacher's rating	41	61	63	72

REPLICATION 1				
	Drew	Neil	Dylan	Mary
**Gender and age at time of inclusion (years, months)**	Girl – 8y 9 m	Boy – 8y, 2 m	Boy – 8y, 11 m	Girl – 10y, 7 m
**Family structure**	Parents divorced	Two‐parent household	Two‐parent household	Parents divorced
**Siblings**	1	3	1	4
**Parent's education level** Number of years of study after graduation	2 (mother); 2 (father)	4 (mother); 2 (father)	0 (mother); unknown (father)	2 (mother); unknown (father)
**Type of injury/diagnosis**	Postinfectious encephalitis (undetermined aetiology)	Severe traumatic brain injury (fall, 3 m)	Fourth ventricle pilocytic astrocytoma	Cerebral anoxia following cardiac arrest
**Glasgow coma scale (+ duration of coma in days)** 13–15: mild; 9–12: moderate; 3–8: severe	N/A	3 (7)	N/A	N/C
**GOS‐E Peds**	6	Severe disability: upper level	Severe disability: lower level	Severe disability: lower level
**Associated impairments**	Cerebellar syndrome	Phonological and speech disorders; written language disorders	Dysarthria; fine motor impairment (distal tremors); dysphagia; diplopia	Bilateral amputation at knee‐level following compartment syndrome; chronic abdominal pain
**Time since injury at time of inclusion (months)**	24 months	13 months	6 months	19 months
**Schooling within rehabilitation department**	Specialised classroom with a small number of students	Specialised classroom with a small number of students	Specialised classroom with a small number of students	Specialised classroom with a small number of students
**BADS‐C** Standard scores; (mean [SD] 10 [3])				
Six parts test	15	10	8	6
Zoo part 1	6	6	8	8
Zoo part 2	3	4	14	6
**Children's cooking task**				
Number of errors/*z* score (compared to age‐matched controls)	101/−6.7	59/−0.3	101/−6.7	23/0.5
Task duration (min)/*z* score (compared to age‐matched controls)	64/−2.9	64/−3	74/−3.9	48/−1.3
**BRIEF questionnaire (before study start)** Global executive composite score (GEC) *T*‐score, (mean [SD] 50 [10]); clinical range cut‐off: *T*‐score ≥ 65				
Parents rating	76	68	70	66
Teacher's rating	69	57	72	93

REPLICATION 2				
	Ron	Adam	Sam	Ian
**Gender and age at time of inclusion (years, months)**	Boy – 16y 10 m	Boy – 12y 10 m	Boy – 13y 6 m	Boy – 16y 1 m
**Family structure**	Two‐parent household	Two‐parent household	Two‐parent household	Parents divorced
**Siblings**	1	0	2	4
**Parent's education level** Number of years of study after graduation	3 (mother); 1 (father)	4 (mother); 2 (father)	2 (mother); 2 (father)	0 (mother); 5 (stepfather)
**Type of injury/diagnosis**	Middle cerebral artery and anterior cerebral artery arterial ischaemic stroke	Severe traumatic brain injury (pedestrian hit by a car)	Severe traumatic brain injury (pedestrian hit by a car)	Parieto‐occipital and interventricular haemorrhagic stroke
**Glasgow coma scale (+ duration of coma in days)** 13–15: mild; 9–12: moderate; 3–8: severe	N/A	7 (22)	9 (22)	N/A
**GOS‐E Peds**	Severe disability: upper level	Severe disability: upper level	Severe disability: lower level	Severe disability: lower level
**Associated impairments**	Aphasia	Word finding difficulties; memory impairment	Behavioural disorders	Right hemiparesis in a right‐handed boy; word finding difficulties
**Time since injury at time of inclusion (months)**	6 months	6 months	6 months	24 months
**Schooling within rehabilitation department**	Specialised classroom with a small number of students	Specialised classroom with a small number of students	Specialised classroom with a small number of students	Specialised classroom with a small number of students
**BADS‐C** Standard scores; (mean [SD] 10 [3]) **BADS (for Ron only)** Standard scores; (mean [SD] 100 [15])				
Six parts test	16	7	5	3
Zoo part 1	0	6	6	8
Zoo part 2		2	1	1
**Children's cooking task**				
Number of errors/*z* score (compared to age‐matched controls)	73/−12	48/−4.5	209/−26.5	63/−9
Task duration (min)/*z* score (compared to age‐matched controls)	58/6.8	44/−1.5	38/−0.8	71/−1.6
**BRIEF questionnaire (before study start)** Global executive composite score (GEC) *T*‐score, (mean [SD] 50 [10]; clinical range cut‐off: *T*‐score ≥ 65)				
Parents rating	51	80	56	72
Teacher's rating	67	67	81	65

*Note*: GOS‐E Peds: Glasgow outcome scale‐extended paediatric version; BADS‐C: Behavioural assessment of the dysexecutive syndrome for children; BADS: Behavioural assessment of the dysexecutive syndrome; BRIEF: Behaviour rating inventory of executive function.

### 3.1 Evolution of transfer goals

**FIGURE 2:**
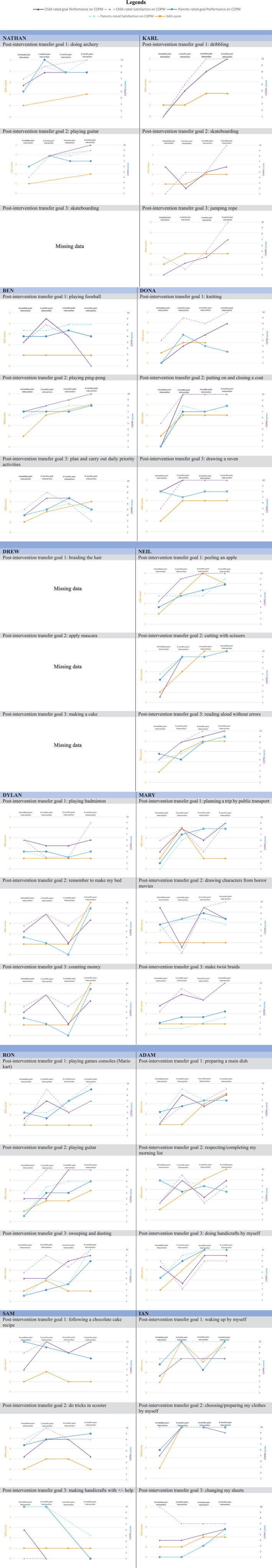
Evolution of goal attainment scale (GAS) scores and Canadian occupational performance measure (COPM) during follow‐up: (1) Meaning of GAS scores: −2: initial level; −1: progress but goal not achieved; 0: goal achieved as expected; +1: goal achieved better than expected; +2: most favourable outcome. (2).COPM: performance and satisfaction are rated by children and parents on a scale of 1 to 10 (1 = not at all able to perform the activity (performance)/not at all satisfied with the way the activity is performed (satisfaction); 10 = perfectly able to perform the activity (performance)/perfectly satisfied with the way the activity is performed (satisfaction). A difference of 2 points between pre‐ and post‐intervention is clinically significant. (3) Graphical representation: evolutions of GAS scores are indicated by an orange line and refer to the *y*‐axis on the left; evolutions of performance (COPM) are indicated by a purple line for children and a blue line for parents; evolutions of satisfaction (COPM) are indicated by a dashed purple line for children and a dashed blue line for parents; COPM scores refer to the *y*‐axis on the right.

Results of the GAS and COPM scores for each of the three post‐intervention transfer goals of the 11 children are summarised in Figure 2. An example of GAS is available in Appendix A.

### 3.2 Visual analysis of GAS scores

At the end of the CO‐OP intervention, the 11 children identified 33 goals they wanted to achieve, 32 of which were measured (Nathan's third transfer goal could not be tested, as mentioned earlier). At 6 months, the results of the GAS show that 22 of 32 goals reached one or more higher levels: 14 goals reached or exceeded the expected level (four goals at level 0; four goals at level 1; six goals at level 2) and eight goals improved without reaching the expected level (i.e. level −1). Two children met or exceeded the expected level for all their goals (Neil and Adam). For Neil, all three goals reached level 0 at 2 months and level +2 at 4 months and then were maintained. Despite observing an improvement in GAS score, four children's performance in these tasks never attained a GAS score of 0 (Karl, Nathan, Sam, and Mary).

### 3.3 Visual analysis of COPM scores by children and their parents

Regarding COPM children's ratings of their perceived performance and satisfaction, the scores of 31 (of the 32) goals were collected (Sam refused to rate handicraft goal at 4‐ and 6‐month follow‐up, reporting that he did not carry out this activity during this period, thinking that it made no sense to rate it). The results showed (1) an important change in perceived performance and satisfaction (≥ 2 positive points change) between the end of the intervention and 6‐month follow‐up for 24 and 25 post‐intervention transfer goals, respectively; (2) no change (or a change of 1 point) in perceived performance for five goals and satisfaction for three goals; and (3) a decrease (≥ 2 negative points change) in perceived performance for two goals and satisfaction for three goals.

Regarding COPM parents' ratings of perceived performance and satisfaction, the scores of 29 (of 32) goals were collected (missing data: Karl's parents' scores). The results showed (1) an important increase (≥ 2 positive points change) in perceived performance and satisfaction for 19 goals between the end of the intervention and 6‐month follow‐up for the two ratings; (2) no change (or a change of 1 point) in perceived performance for seven goals and satisfaction for eight goals; (3) a decrease (≥ 2 negative points change) in perceived performance for three goals and satisfaction for two goals.

### 3.4 Statistical analysis of the evolution of the GAS scores and parent‐ and participant‐rated COPM performance and satisfaction scores over time

Table 4 present the results of the Friedman test, indicating a statistically significant change in mean GAS scores for participants' goals between T0 and T3. Similarly, the differences of the mean three post‐intervention transfer goals participant‐ and parent‐rated performance and satisfaction COPM scores between T0 and T3 were all statistically significant.

**TABLE 4:** Evolution of GAS and COPM scores of transfer goals over time from immediate post‐intervention (T0) to 6‐month post‐intervention (T3).

	Post‐intervention									
	*Immediate (T0)*		*2‐months (T1)*		*4‐months (T2)*		*6‐months (T3)*		Friedman test	
	*n*	Median (min; max)	*n*	Median (min; max)	*n*	Median (min; max)	*N*	Median (min; max)	*df*	Chi‐square^a^
**GAS score**										
***All children***	32	−2 (−2; −2)	30	−1 (−2; 2)	30	−1 (−2; 2)	30	−1 (−2; 2)	3	38.812***
***Medium to high parents' involvement***	21	−2 (−2; −2)	21	−1 (−2; 2)	21	0 (−2; 2)	21	0 (−2; 2)	3	35.619***
***Poor or no parents' involvement***	11	−2 (−2; −2)	9	−2 (−2; 1)	9	−2 (−2; −1)	11	−2 (−2; −1)	3	6.143
**COPM**										
***All participants***										
*Child's performance*	32	5 (1; 9)	32	8 (1; 10)	32	7.5 (1; 10)	32	8 (1; 10)	3	34.097***
*Child's satisfaction*	32	5 (1; 10)	32	8 (1; 10)	32	7.5 (1; 10)	32	10 (1; 10)	3	36.358***
*Parents' performance*	28	5 (1; 10)	28	6.5 (1; 10)	28	7 (1; 10)	28	8 (1; 10)	3	22.925***
*Parents' satisfaction*	29	5 (1; 10)	29	7 (1; 10)	29	8 (1; 10)	29	8 (3; 10)	3	27.387***
**COPM**										
***Medium to high parents' involvement***										
*Child's performance*	21	5 (1; 8)	21	8 (4; 10)	21	7 (3; 10)	21	8 (1; 10)	3	27.361***
*Child’ satisfaction*	21	5 (3; 10)	21	8 (3; 10)	21	7 (3; 10)	21	9 (1; 10)	3	19.083***
*Parents' performance*	20	5 (1; 8)	20	6 (1; 10)	20	7 (1; 10)	20	8 (3; 10)	3	21.368***
*Parents' satisfaction*	21	5 (1; 10)	21	7 (1; 10)	21	7 (1; 10)	21	8 (3; 10)	3	19.581***
***Poor or no parents' involvement***										
*Child's performance*	11	6 (1; 9)	11	8 (1; 10)	11	8 (1; 9)	10	8.5 (5; 10)	3	11.000
*Child's satisfaction*	11	7 (1; 8)	11	9 (1; 10)	11	9 (1; 10)	10	10 (8; 10)	3	19.022***
*Parents' performance*	8	6 (2; 10)	8	8 (4; 10)	8	8 (4; 10)	8	7 (1; 9)	3	5.913
*Parents' satisfaction*	8	5.5 (1; 10)	8	8.5 (2; 10)	8	8.0 (3; 10)	8	8.5 (4; 10)	3	9.310

^a^ Chi‐square correspond to difference between T0 and T3.

*** *p* < .001.

### 3.5 Exploration of the possible influence of the degree of parents' involvement in CO‐OP on children's attainment of transfer goals

Of the 11 children, the parents of seven were classified as being in the medium to high involvement category during the CO‐OP process, whereas the parents of four children were classified in the poor or no involvement category.

**FIGURE 3:**
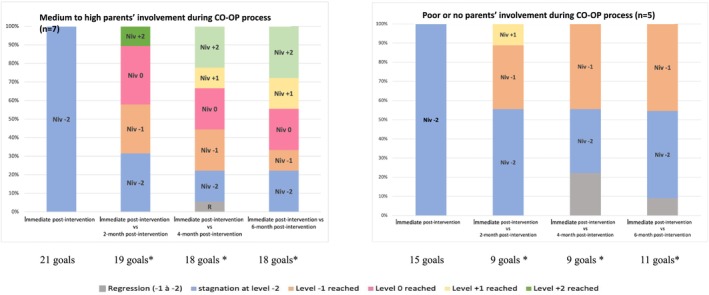
Evolution of goal attainment scale (GAS) scores of transfer goals of children depending on parents' involvement. *Missing data for some goals.

The children, whose parents were classified as having medium to high involvement throughout the CO‐OP process, showed a variation on goal achievement at 6‐month follow‐up: a third of goals (14/21) were attained, at or exceeded the expected level of the GAS, progress was noted on three (attained level −1), and no progress was seen in four goals (remained at level −2) (Figure 3). However, children whose parents were classified as having poor or no involvement during CO‐OP process showed less variation on goal achievement, as little or no progress in their performance was noted (reached −1 or remained at level −2) or reached −1 for all goals, that is, none of those children had reached the expected level. The only exception was on one occasion at 2‐month post‐intervention for one of Mary's goals (planning a trip by public transport) where a GAS score of 1 was attained.

These results are in line with those of the Friedman test where statistically significant *(p* < 0.001) change in GAS scores was obtained for children whose parents were classified as having ‘medium to high’ involvement. This contrasts with a non‐statistically significant change in GAS scores for the children whose parents were classified as having ‘poor or no’ involvement. Similarly, a statistically significant *(p* < 0.001) change in both COPM scores (perceived performance and satisfaction) was obtained for both children and parents in the category ‘medium to high involvement’; while a non‐statistically significant change in COPM perceived performance scores for both children and parents in the category ‘poor or no involvement’.

### 3.6 Association between G&T scale scores and changes in GAS scores

All correlation coefficients were very small (range from 0.056 to 0.164) and non‐significant. These results show no association between the GAS (level of attainment) of the new transfer goals and the degree of similarity (measured by G&T scale) between the tasks trained during CO‐OP intervention and the new transfer goals.

## 4 DISCUSSION

The aim of this study was to (1) assess whether following CO‐OP intervention, without any further specific intervention, improvement was noted in the occupational performance of new goals (transfer goals) set at the end of the CO‐OP intervention phase; (2) study the possible influence of the involvement of parents in CO‐OP on children's attainment of transfer goals; and (3) determine if the degree of difference between trained and untrained transfer goals is associated with the achievement of the transfer goals. The findings suggest that children continued to apply the strategies and skills learned through CO‐OP intervention to their new untrained transfer goals. In addition, results showed that children whose parents were classified as ‘being involved’ made important progress in goal attainment, while children whose parents were classified as having ‘poor or no involvement’ did not. Finally, no association was found between the proximity of the tasks (determined using the G&T scale) and whether or not transfer goals were achieved.

According to the visual analysis of the GAS graphs (Figure 2), at 6‐month follow‐up, over two thirds of the new untrained transfer goals (22 of 32 goals), selected by the 11 children at the end of the intervention phase, showed important improvement, with almost half (14 goals) reaching or exceeding the expected level. These observations were confirmed by statistical analyses where significant improvement of GAS scores over time was found for the untrained transfer goals. To the best of our knowledge, our study is the only one to date to study transfer by setting new goals set at the end of the CO‐OP intervention and measured using an objective measure (GAS), supplemented by a perceived self‐report measure by the parents and children (COPM).

Other CO‐OP studies explored transfer by identifying control goals concurrent with the CO‐OP intervention. Hunt et al. (2019) reported on transfer of learning in youth with ABI following a CO‐OP intervention. The study determined transfer by identifying control untrained goals concurrent with the CO‐OP intervention and measured changes using the COPM. They reported perceived clinically significant changes on untrained goals, suggesting preliminary evidence of transfer. Dawson et al. (2009) explored the effectiveness of CO‐OP with three adults with EF deficits following head injury. The positive changes in the COPM for both trained and untrained goals also suggest that CO‐OP can encourage the transfer of strategy use to new tasks. The DEX (DysEXecutive) questionnaire (a daily life measure of executive dysfunction) (Burgess et al., 1998) was also used in this study. Pre‐post (CO‐OP intervention) DEX score changes also suggested a transfer effect. Follow‐up scores (after 3 months) suggest that changes were maintained over time, as found in the current study.

Öhrvall et al. (2023) also demonstrated that, in children with cerebral palsy or spina bifida (*n* = 34) with EF deficits, CO‐OP intervention can lead to a transfer effect over time (follow‐up of 3 months after an 11‐session intervention) of untrained concurrent control goals (*n* = 1). To analyse the presence or absence of transfer, the authors also used the COPM, as well as the Performance Quality Rating Scale (PQRS), an observational measure of performance quality (Martini et al., 2015). They measured changes in concurrent control (transfer) goals before the intervention, immediately post‐intervention and then after 3 months. They found that children's transfer goals changed more significantly at the 3‐month follow‐up than at post‐intervention. The authors hypothesised that children need time to learn to adopt the CO‐OP approach and engage in metacognitive thinking in new situations. The result of the qualitative analysis of the parents/caregivers semi‐structured interviews in our study supports this finding (Dietrich et al., 2024). Indeed, one of the themes that emerged from this analysis was that CO‐OP mastery requires time and practice, and its use evolves over time.

No association between the GAS (level of attainment) of the new transfer goals and the G&T score (degree of similarity between the trained tasks during CO‐OP intervention and the new transfer task) was found in our study. This suggests that improvement in untrained transfer goals was not related to the similarity of those new goals with the goals that were trained during the CO‐OP intervention. These results suggest that transfer can occur despite minimal similarity, contrary to Thorndike's Identical Elements Theory (IET) for understanding transfer, where the degree of transfer between the intervention task and the new task depends on the number of similar elements (Thorndike, 2013). Rather, these results suggest that the transfer process potentially goes beyond consideration of the context and pattern of the tasks being compared. Indeed, our results are in line with those of Houldin (2018), who also studied the link between the degree of similarity between two activities and the degree of transfer, finding no association.

The findings of our study point to the important influence parent involvement may have had in the CO‐OP process on the change of GAS scores of the untrained transfer goals. Indeed, children with parents demonstrating medium to high involvement showed a greater change across time in GAS scores for the transfer goals than children whose parents with poor or no involvement. These results reflect the CO‐OP principle of the importance of ‘significant other involvement’ (Polatajko & Mandich, 2004) and are in line with studies showing that parental involvement is an important factor to consider when implementing interventions in paediatric rehabilitation (Laatsch et al., 2020). These results corroborate those obtained by Capistran and Martini (2016), who investigated whether CO‐OP improved performance on a concurrent untrained task (inter‐task transfer) in four children with developmental coordination disorders using a single subject design. While authors found that CO‐OP is effective in improving skills in trained tasks, improvement in untrained tasks was variable. Authors explain that for children where transfer did not occur, parents reported limited or non‐existent use of the CO‐OP global strategy, as well as inconsistent completion of practice assignments outside of intervention session. They suggest that differences in parental support might have enabled some children to become more familiar with CO‐OP and thus more easily apply it to their untrained goals. Our results support this proposition.

It is difficult to pinpoint exactly why some parents were highly involved in the CO‐OP process, while others had poor or no involvement. One of the many possibilities may be that CO‐OP and its benefits may not have been properly explained to or understood by the parents. However, this is unlikely, as the same principal investigator explained the CO‐OP principles to all patients and families in the study. Furthermore, the occupational therapist responsible for the CO‐OP intervention exchanged with parents every week about the evolution of the CO‐OP sessions (or at least passed on the information to the parents if they were not available for a face‐to‐face discussion). Another more likely explanation may be that it was challenging for some parents to incorporate practice or elements of CO‐OP in their complex daily family routine in the sub‐acute phase following their child's brain injury. In our qualitative study, parents reported difficulty in integrating practice into the busy family routine and among other responsibilities (Dietrich et al., 2024). Indeed, this was a challenge also reported by parents of children with developmental coordination disorder in several studies (Capistran et al., 2015; Martini et al., 2021). Similarly, Jenkin et al. (2023) refer to this aspect as ‘juggling family life’ in their study exploring experiences of family‐centred service during rehabilitation from the perspectives of parents/caregivers, siblings, and children/adolescents with ABI. Discussion with parents and care partners to confirm their understanding of the approach as well as how to facilitate the incorporation of its elements in their daily routine might be helpful to facilitate parent involvement.

### 4.1 Strengths and limitations of the study

A strength of this quasi‐experimental study is the measurement over four time periods, including at 6‐month post‐intervention follow‐up, which is longer than previously published studies. The protocol, with three untrained transfer goals chosen by children, and the use of both objective (GAS) and perceived (COPM) measures, provided a rather robust framework for determining learning transfer. This ‘untrained task’ approach is recommended by Geusgens et al. (2007), as these transfer goals accurately allowed assessing true generalisation and transfer of benefits of the CO‐OP intervention in everyday life. Another strength is the establishment of inter‐rater reliability for the GAS, the G&T scale ratings, as well as for the categorisation of children according to parental involvement by independent raters. Lastly, the GAS videos were randomised to prevent GAS rater bias.

There are also several limitations to this study. First, there was missing data for the GAS and COPM scores, particularly for the parents' COPM scores. Despite these missing data, statistical analyses could still be conducted effectively and results interpreted appropriately. Another limitation is that it was not possible to videotape the children achieving their new goals in their real‐life environments (i.e. home and school) because of practical and organisational constraints. This may have restricted the possibilities of transfer with respect to the context rating of the G&T scale. Nonetheless, despite this restriction, evidence of transfer was obtained.

## 5 CONCLUSION

This study demonstrates that CO‐OP effectively facilitates the transfer of acquired skills to new untrained activities, even after the intervention concludes. To our knowledge, it is the first study to examine the transfer of skills to untrained goals in such detail, rigour, and originality. Notably, no significant association was found between the similarity of trained and untrained tasks and the achievement of transfer goals. Additionally, this study highlights the potential importance of parent involvement, finding that it might be a critical factor in promoting skill transfer. These findings align with prior research emphasising the pivotal role of parental engagement in interventions aimed at enhancing children's activities and participation. Future studies should focus on identifying the facilitators and obstacles to parental involvement, to optimise intervention delivery and improve outcomes for children.

## AUTHOR CONTRIBUTIONS

H. Lebrault, R. Martini, and M. Chevignard contributed to this study's conception. H. Lebrault conducted data collection, with the help of the occupational therapists who led the CO‐OP sessions. C. Vezinat completed the data analysis, with the support from the other authors and in particular of H. Câmara‐Costa. C. Vezinat and H. Lebrault wrote the manuscript. All the authors critically revised the work for submission and agreed to be accountable for all aspects of the work.

## CONFLICT OF INTEREST STATEMENT

The authors report no conflicts of interest.

## Data Availability

The data that support the findings of this study are available from the corresponding author upon reasonable request.

## APPENDIX A Example of a GAS scale

**Table:**

GAS 1 bis ‐ dribbling with a soccer ball	
−2 Initial level (immediate post‐intervention, T0)	Ball at foot, shoots the ball and lets it slip away by throwing it too far.
**−1**	Can perform an uncontested leg pass at least 4 times out of 10 (= without opponent).
**0** Expected level at 6‐month follow‐up (T3)	Can perform an uncontested leg pass at least 7 times out of 10 (= without opponent).
**+1**	Can perform a leg pass at least 7 times out of 10, in front of an opponent, but misjudges the timing and the opponent regains possession most of the time.
**+2**	Performs a leg pass at least 7 times out of 10, facing an opponent, and correctly assesses the timing of the movement; the opponent intercepts the ball less than 50% of the time.

The initial level for all GAS was established on the basis of the participant's initial performance, through direct observation and video analysis of the participant's performance by the therapist.	
**Data collection method:** ‐ 1 time at the end of the intervention, then during follow‐up (2‐, 4‐, and 6‐month post‐intervention). ‐ location: 1st floor corridor ‐ warm‐up, then the participant indicates when he/she is ready for the test ‐ opposing player: occupational therapist ‐ person responsible for collecting GAS: occupational therapist ‐ videos	
